# Resting-state EEG topographies: Reliable and sensitive signatures of unilateral spatial neglect

**DOI:** 10.1016/j.nicl.2020.102237

**Published:** 2020-03-05

**Authors:** Elvira Pirondini, Nurit Goldshuv-Ezra, Nofya Zinger, Juliane Britz, Nachum Soroker, Leon Y. Deouell, Dimitri Van De Ville

**Affiliations:** a Institute of Bioengineering/Center for Neuroprosthetics, Ecole Polytechnique Federale de Lausanne (EPFL), Lausanne, Switzerland; b Department of Radiology and Medical Informatics, University of Geneva, Geneva, Switzerland; c Department of Neurological Rehabilitation, Loewenstein Rehabilitation Hospital, Raanana, Israel; d Evoked Potentials Laboratory, Technion – Israel Institute of Technology, Haifa, Israel; e Department of Psychology and Edmond and Lily Safra Center for Brain Sciences (ELSC), The Hebrew University of Jerusalem, Israel; f Department of Psychology and Neurology Unit, Medicine Section, Faculty of Science and Medicine, University of Fribourg, Fribourg 1700, Switzerland; g Sackler Faculty of Medicine, Tel Aviv University, Tel Aviv, Israel

**Keywords:** Unilateral spatial neglect, Stroke, Rehabilitation, Outcome measurement, Computer-enhanced measurement, EEG analysis, EEG topography features, Resting-state EEG biomarkers, Machine learning

## Abstract

Theoretical advances in the neurosciences are leading to the development of an increasing number of proposed interventions for the enhancement of functional recovery after brain damage. Integration of these novel approaches in clinical practice depends on the availability of reliable, simple, and sensitive biomarkers of impairment level and extent of recovery, to enable an informed clinical-decision process. However, the neuropsychological tests currently in use do not tap into the complex neural re-organization process that occurs after brain insult and its modulation by treatment. Here we show that topographical analysis of resting-state electroencephalography (rsEEG) patterns using singular value decomposition (SVD) could be used to capture these processes. In two groups of subacute stroke patients, we show reliable detection of deviant neurophysiological patterns over repeated measurement sessions on separate days. These patterns generalized across patients groups. Additionally, they maintained a significant association with ipsilesional attention bias, discriminating patients with spatial neglect of different severity levels. The sensitivity and reliability of these rsEEG topographical analyses support their use as a tool for monitoring natural and treatment-induced recovery in the rehabilitation process.

## Highlights

• EEG-SVD topographies correlate with signatures of stroke.
• They are robust over repeated measurements and new patient samples.
• They correlate with the major signs of unilateral spatial neglect.

## 1 Introduction

Stroke is the most frequent neurological disease in western societies (Truelsen et al., 2006; Vaartjes et al., 2008). For survivors, the consequences are frequently dire, with severe impact on their ability to accomplish activities of daily living independently and on their quality of life, as well as on the related healthcare costs for families and societies. This triggered a surge of interest in the design of novel therapeutic interventions to enhance the rehabilitation outcome. The translation of such novel protocols into daily clinical practice depends on the ability of the clinician to discern levels of impairment and monitor the efficacy of treatment with simple and robust methods (Coscia et al., 2019).

In clinical rehabilitation practice, treatment assessment is guided largely by functional outcome measures defined at the group level. Such measures are typically based on performance of standardized behavioral tasks and more recently on computer-based measures of behavioral deficits and their dynamics during functional recovery (Bosecker et al., 2010; Panarese et al., 2016; Bonato and Deouell, 2013). However, both conventional and computer-based assessment tools are sensitive to both restoration and compensation processes, making it difficult to isolate restitution success at the level of more discrete brain functions. This limitation is of particular significance when there is need to assess the impact of experimental interventions aimed to restitute impaired function through facilitation of adaptive structure-function re-mapping processes (e.g., protocols employing non-invasive brain stimulation in conjunction with behavioral therapies Lefaucheur et al., 2014; Lefaucheur et al., 2017; Hummel et al., 2008; Kang et al., 2016; Takeuchi et al., 2005). The success rate of such interventions is naturally affected by inter-personal variance in the extent to which different brain structures and neurophysiological operations are impaired by the stroke. Therefore, in addition to measures targeting the impairment level from a behavioral perspective, there is a need for new diagnostic tools that can capture dynamic changes at the physiological level, allowing the clinician to monitor, inform, and adapt interventions, in a personalized manner. Such diagnostic tools should: *i)* inform the impairment level of a patient in a reliable, objective manner; *ii)* show their reliability over repeated assessment sessions when performance level is stable.

A number of studies showed that recording of spontaneous brain activity could be of clinical value, providing biomarkers^2^ to delineate responses to treatment at the neurophysiological level (Woo et al., 2017; Rossini et al., 2003; Allali et al., 2018). These so-called “resting-state” biomarkers can be obtained using functional magnetic resonance imagining (fMRI) (Corbetta et al., 2005; Preti et al., 2017) or EEG-based protocols (Allali et al., 2018). Thanks to its simplicity and ease of employment within the rehabilitation setting, EEG-based analysis is of particular appeal. Abnormalities in EEG spectral composition and in functional connectivity at rest have been associated with behavioral deficits post-stroke (Finnigan et al., 2007; Laaksonen et al., 2013; Giaquinto et al., 1994) and have been shown to be predictive of long-term post-stroke outcome (Zhang et al., 2013; Aminov et al., 2017; Sheorajpanday et al., 2011; Finnigan and van Putten, 2013).

We have shown that dynamics in the organization of resting-state EEG (rsEEG) topographies correlate with motor control strategies, offering a powerful tool for the characterization of brain activity following stroke (Pirondini et al., 2017; Minguillon et al., 2014; Pirondini et al., 2018). Here, we applied this approach to a population of post-stroke subjects with different degrees of unilateral spatial neglect (USN). USN is a frequent sequel of right hemisphere lesions, which gravely hinders patients’ rehabilitation (Katz et al., 1999). Its most typical characteristic is failure to orient to, report or respond to visual, somatosensory, and auditory stimuli presented in the contralesional space (Parton et al., 2004). The great majority of patients suffering from USN are unaware of their deficit, further complicating rehabilitation.

Here, we aimed to assess whether topographical analysis of rsEEG patterns would represent an objective diagnostic tool in USN. We measured brain activity at rest using EEG in two cohorts of sub-acute stroke patients (total number of patients - 33) and evaluated the reliability of these EEG topographical analyses by testing them over several recording sessions, on separate days. During each session, lateralized bias in spatial attention was behaviorally measured using the Starry-Night test (SNT), a computer-based visual-search task that exhibits higher sensitivity in detection of deficits in spatial attention compared to traditional paper and pencil tests (Sacher et al., 2004; Deouell et al., 2005). We used spontaneous spatially-distributed activity (i.e., rsEEG topographies captured using singular value decomposition) in the slow (delta and theta) and high (alpha and beta) frequency bands to discriminate patients from matched healthy control subjects, and to distinguish different levels of spatial neglect. Our approach was able to robustly distinguish patients from healthy controls based on the EEG alone. Post-stroke EEG abnormalities correlated strongly with visual-search abnormalities detected by the SNT, allowing to discriminate patients with different severity levels of USN. The sensitivity of the rsEEG topography-based patterns was held across repeated testing sessions, emphasizing the potential of spontaneous brain activity to be used clinically for longitudinal monitoring.

## 2 Materials and method

### 2.1 Participants

In total, 216 EEG recordings from 33 first-event stroke patients in the subacute period (1–3 months after onset) and 6 healthy controls were analyzed. Patients were recruited in two different studies, thus creating two groups: group #1–20 patients, 15 with right- and 5 with left-hemisphere damage (RHD, LHD); group #2 - 13 RHD patients. Patients of the two groups underwent the same tests (see Section 2.4 *Experimental protocol*), whose data were included in this study. In addition to the tests reported here, experimental sessions of group #1 comprised of resting-state EEG recording with eyes closed for 6 min, a modified version of the SNT, and a visual evoked potential task. Instead, patients of group #2 performed rehabilitation sessions of EEG neurofeedback. These recordings were out of the scope of this work and, therefore, the data were not included in this study. Patients in each group were recruited from sequential admissions to the Department of Neurological Rehabilitation at the Loewenstein Hospital (LRH), Raanana, Israel. Inclusion criteria were similar in both groups: first-ever unilateral ischemic or hemorrhagic hemispheric stroke; stable clinical and metabolic state at the time of testing; no history of neurological or psychiatric disease; lack of visual field defects; cognitive and linguistic state enabling comprehension of task requirements. Healthy control participants were recruited from either the hospital personnel or patients’ family members. The LRH Helsinki committee approved the study and written informed consent was obtained from all subjects (see Supplementary Table 1 for patients’ details).

### 2.2 Lesion location and volume measurement

For each patient, follow-up computerized tomography (CT) was conducted to calculate lesion location and volume during the rehabilitation period at the Loewenstein Rehabilitation Hospital. Lesions were manually outlined on the digitized CTs using the MEDx software (Medical-Numerics, Sterling, VA, USA). Each scan was analyzed using the Analysis of Brain Lesions (ABLe) module implemented in Medx. Anatomical structures in the normalized brain were reported by using the Automated Anatomical Labeling (AAL) atlas (Tzourio-Mazoyer et al., 2002) and the amount of lesioned tissue was quantified in those standard structures as described in Haramati et al. (2008).

### 2.3 Paper and pencil standardized neglect tests (PPT)

In addition to the SNT used in both groups (see below), group #2 was also tested using the standardized Behavioral Inattention Test (BIT) (Wilson et al., 1987) as well as with an extended Line Bisection Task (LB) (Anderson, 1996) and the Mesulam and Weintraub Cancellation Task (MWCT) (Weintraub and Mesulam, 1987) (see Supplementary Table 2).

The BIT is a standardized test battery for neglect in visual modality and includes three distinct target cancellation tasks (lines, letters, stars); figure and shape copying; line bisection; and representational drawing. Patients were presented with all subtests at midline and were asked to inform the experimenter as soon as they accomplished each subtest. The order of the subtest was similar for all participants. Maximum total score is 146 and cut-off for normality is 129 points (Wilson et al., 1987). Cut-off for normality in the Star Cancellation (SC) subtest, i.e., the most sensitive of the subtests, is 52 (maximal score: 54) (Halligan et al., 1990).

During the extended LB test, subjects were presented test sheets and were asked to mark the center of horizontal black lines on A4 sheets (21.6 × 27.9 cm) of white paper. The pencil was required to be held with the ipsilesional right hand. The patients were asked to bisect 10 lines for each length (36 mm, 90 mm, and 180 mm) provided in ostensibly random order. The deviation (in mms) of the point marked by the patient (subjective midpoint) from the true midpoint of the line was measured. The cumulative deviation from the true center was calculated for all lines. Rightward and leftward deviations were assigned positive and negative signifiers respectively (Anderson, 1996). Scores were considered pathological if the deviation was greater than 10%.

For the MWCT, subjects were given an A4 sheet of paper containing randomly arranged shapes. Each quadrant of the page contained 90 shapes, 15 of which were target stimuli. The midline of the test sheet was aligned with the midline of the subject's body and the subject was asked to circle all the targets. A time limit was not imposed on the patients, however the time of completion was measured and noted. The numbers of targets circled on the right and on the left were counted (Weintraub and Mesulam, 1987). The correct number of targets for each side was 30.

### 2.4 Experimental protocol

In order to evaluate the reliability of the EEG-derived patterns across times of day and across days, we tested each participant several times. Specifically, patients of group #1 and healthy control subjects were tested 3 times per day (morning, noon, late afternoon) in 2 different days about one week apart, and patients of group #2 were tested once per day, over 10 different days, about 2 days apart. In both groups, testing of patients started during the subacute period (39±21 and 54±21 days post onset, for patients of groups #1 and #2, respectively, see Supplementary Table 1). Some patients did not complete all testing sessions due to occasional conflicts with treatment schedules or family visits (see Supplementary Tables 3 and 4 for more details). Testing sessions comprised of resting-state EEG recording with eyes open for 6 min, followed by visual-search testing with the SNT (see Section 2.5) (Sacher et al., 2004; Deouell et al., 2005).

### 2.5 Dynamic spatial attention testing (Starry Night Test)

Spatial attention was assessed using the ”starry night test” (SNT) (Deouell et al., 2005), a computerized search task measuring reaction time and detection accuracy (hit rate) for target visual stimuli. The SNT was operated in the current study as follows: the patient was seated in front of a computer screen (16º and 12º visual angels, in horizontal and vertical dimensions, respectively), which was virtually divided into a 5 × 5 matrix for the healthy subjects and patients of group #1, and a 7 × 7 matrix for patients of group #2. On each trial, a single target stimulus was presented in a different position on the computer screen, among distracters differing from it in shape and color, thus enabling detection by spread attention through ‘pop-out’ mechanism (all the distracters were of similar shape, size and color). Every trial began with the distracter in each cell being either randomly visible or invisible. Every 50–250 ms (random interval selection) one cell of the matrix was chosen and the status of its distracter was toggled so that if it was invisible it became visible and vice versa. The exact location of the distracter within the cell was varied randomly. 700 to 2100 ms from the beginning of the trial the target stimulus appeared inside one of the virtual cells while the distracters continued to flicker. Right-hemisphere damaged patients and healthy subjects were instructed to hit a specified key on a serial response box with the index finger of the right hand as soon as the target stimulus was detected. Left hemisphere damaged patients used the left hand in order to avoid possible right hand paresis as a confound factor. If the participant pressed the key before the actual appearance of the target, the response was defined as a false alarm. If the participant did not respond within 3000 ms of the appearance of the target, a miss trial was recorded. Miss trials were not replaced. Altogether, the target appeared 4 times in each of the 25 cells for healthy subjects and patients of group #1, and 3 times in each of the 49 cells for patients of group #2. The target stimuli were 4 mm solid blue squares, whereas the distracter stimuli were 2 mm solid red circles. The SNT allows a detailed statistical assessment of spatial gradients of performance, as well as evaluation of changes in these gradients across time, both at individual and group level. The independent variable is the horizontal location of the target stimulus on the screen and the dependent variables are the reaction time (RT) to the target stimulus and the hit rate (HR). The patient was monitored via a video camera facing the patient and the test was interrupted if the participant slumbered or a constant gaze shift was noticed. The experiment was controlled by E-prime 2 (Psychology software tools, Pennsylvania, USA). Prior to the initiation of the test, the participants were shown examples of the distracter and target stimuli and it was confirmed that they could tell them apart. See Deouell et al. (2005) for more details on the SNT.

### 2.6 Computation of behavioral measures in the SNT

For each participant and every session, we computed several parameters in order to evaluate behavioral differences across patients and healthy controls. Specifically, we assessed the percentage (over session) of hit targets (*Hit*) to evaluate the capacity of the patients to discern targets. Left- and right-sided targets (omitting the central column) were divided into two equal spaces. We computed left and right mean reaction time (LMRT, RMRT) and RT variance (LVRT, RVRT). For both left and right hemispaces, RT deviating by more than 2 standard deviations from the mean over all the targets in that particular hemispace were discarded, to reduce the influence of outlier events (Anderson et al., 2000) (percentage of outliers over trails: 1.81 ± 0.13 averaged ± SEM over subjects, see Supplementary Table 5). No significant differences were found in the number of outliers between patients and healthy controls (one-tailed, non-paired *t*-test with heteroschedasticity (α=0.05)). Finally, we computed two indices to further specify performance differences between right and left visual search: Laterality Index (LI) $LI=\frac{\left(RMRT-LMRT\right)}{\left(RMRT+LMRT\right)}$ and $F=\frac{LVRT/|LMRT|}{RVRT/|RMRT|}$. LI would be positive (negative) if RTs of left-sided (right-sided) targets would be shorter than those of right-sided (left-sided) targets. The F value is the ratio of the coefficients of variation (variance normalized by the mean – CV) of left- versus right-sided RTs and would be < 1 if left-sided CVs were lower than right-sided CVs.

### 2.7 EEG data acquisition and pre-processing

An Active II EEG system (Biosemi, Amsterdam) with 32 pre-amplified (active) EEG channels was used for testing healthy subjects and patients of group #1 and 64 channels for testing patients of group #2. The electrodes were embedded in an elastic cap based on the extended 10–20 system (see https://www.biosemi.com/pics/cap_32_layout_medium.jpg,https://www.biosemi.com/pics/cap_64_layout_medium.jpg for electrode layout). Four active EOG electrodes (two above and below the right eye and two next to the lateral canthi of both eyes) were also recorded in all participants in order to detect eye-movement artifacts. Sampling rate was 1024 Hz and 256 Hz for groups #1 and #2, respectively. The EEG data of group #2 was down-sampled offline to the same 32 channels of group #1. A low pass filter with a cutoff of 1/5 of the sampling rate was applied during recording to avoid aliasing of high frequencies. EEG data was pre-processed offline using MATLAB (MathWorks, Natick MA) and EEGLAB toolbox (Delorme and Makeig, 2004). The raw EEG data was filtered (1 Hz to 40 Hz, Butterworth zero-phase 8th order IIR filter (Dipietro et al., 2014; Van de Ville et al., 2010)) and down-sampled to 128 Hz. Noisy electrodes were interpolated using spherical spline interpolation and, then, the data was re-referenced to a common average. ‘Eye blink’ and ‘eye movements’ artifacts were removed by Independent Component Analysis (ICA) (Delorme and Makeig, 2004). The data was then visually inspected to remove periods contaminated by artifacts (i.e., amplitude > 80 μV) and the remaining data was concatenated.

### 2.8 Computation of rsEEG topography measures

Different techniques have been proposed for extracting dominant topographies from multichannel EEG signals (Michel et al., 2009; König and Gianotti, 2009; Higashi et al., 2014). For this aim, here we employed singular value decomposition (SVD) (Pirondini et al., 2018; Sivakumar et al., 2016). The SVD of a real matrix is a factorization of the form $E=MSN^{⊺}$. In our case, the $E\in \;R^{C}\times R^{T}$ real matrix was the matrix of the pre-processed EEG signals concatenated across sessions and participants in time. This concatenation in time assumes that all subjects have similar spatial map per component. We tested and demonstrated that this was indeed the case (see Supplementary Figure 1A-B). *C* was equal to 32 EEG channels and *T*  was the total time of recordings summed over sessions and participants. The left-singular vectors of $M\in R^{C}\times R^{C}$ represented the group-level EEG-SVD topographical maps. They were ranked according to their non-zero singular values, which are the diagonal values of *S*. The right-singular vectors $N\in R^{C}\times R^{T}$ represented the group-level time courses of the corresponding topographical maps. In order to obtain the individual (i.e., for each participant) time courses, the EEG activity of each individual for each session was projected on the group-level EEG-SVD topographical maps corresponding to 80% of the explained variance. For instance, the time courses for the first {1, ⋅⋅⋅, *c*} SVD components for one subject was obtained as ${\tilde{E}}_{1}=M_{1,\ldots ,c}^{T}E_{1}$. The explained variance was computed from the singular values as: $VAR_{i}=\sum_{j}^{C}\frac{S_{j}^{2}}{\sum S_{j}^{2}}*100$.

Reproducibility of the EEG-SVD group-level topographies was assessed by split-half reproducibility analysis. Specifically, the original set of 37 subjects (i.e., healthy controls and patients of the two groups) was randomly split into 2 groups. Ten different random splits were generated. Subsequently, for each split, EEG-SVD topographies were computed for each group concatenating the data of all subjects within the group. EEG-SVD topographies obtained from the two groups were then matched using Hungarian algorithm (Munkres, 1957), and Pearson correlation was computed for the first 5 components (i.e., components corresponding to 80% of the explained variance) between the two groups.

For each time course of each subject, a time-frequency representation was calculated from 1 to 64 Hz by convolving the signals with a complex-valued Morlet wavelet with 3 cycles. Time-frequency representations of power were calculated as the squared magnitude of the complex wavelet-transformed data. We then considered coefficients of variation (i.e., the ratio between variance and mean spectral power over time) for four typical frequency bands (i.e., δ: 1–3.8 Hz; θ: 4.1–7.6 Hz; α: 8.2–12.4 Hz; β: 15.3–30.6 Hz).

### 2.9 Comparison of behavioral and rsEEG measures between patients and healthy subjects

In order to establish whether the behavioral measures in the SNT and the rsEEG topography-based patterns could provide reliable signatures of stroke, we first compared each behavioral measure and the coefficient of variation (CV) of each frequency band and EEG-SVD component between patients and healthy subjects using a one-tailed, non-paired *t*-test with heteroschedasticity (α=0.05).

Next, we employed a Bayesian classifier, specifically, a Linear Discriminant Analysis (LDA) (Fisher, 1936), to mathematically quantify the separability between right-hemisphere damaged (RHD) patients and healthy controls and to assess the most informative behavioral and brain measures. We built a two-class LDA classifier (accounting for different covariance matrices for each class) for behavioral measures and for CVs of the EEG-SVD components separately. To rank the features of the classifier, we calculated the discrimination power for the two classes (i.e., RHD patients and healthy controls) for each feature separately, using a two-sample Mann-Whitney test. Next, we ranked the features by their absolute standardized u-statistic obtained from this test. In the next stage, we sequentially added feature after feature and tested classification accuracy by performing a leave-one-out cross-validation, leaving out, for each fold, all the sessions of one subject (either healthy control or patient) for training, and then testing on the left-out subject. Decoding accuracy values were then averaged over cross-validation folds. Receiver operating characteristic (ROC) curves and confusion matrices were computed considering all cross-validations folds. The ROC analysis allows assessing the trade-off between sensitivity and specificity. We then calculated the area under the ROC curve (AUC), which is a direct measure of the diagnostic power of the test. An AUC of 1.0 indicates a perfect classification: all patients are classified as such while none of the healthy subjects is diagnosed as being a patient. Finally, we chose the combined classifier with the highest performance.

In order to assess the statistical significance of the AUC values, we built 1000 classifiers with randomly assigned labels at each permutation. We estimated AUC value for each permutation. The threshold for significance was defined as the 95th percentile of this single tail null distribution.

It is important to highlight that in order to avoid over-fitting, new group-level topographical maps were obtained for each cross-validation fold by concatenating the EEG data of all participants and sessions excluding the sessions of the left-out subject. The EEG activity of the left-out subject was then projected into the EEG-SVD components that were obtained concatenating the other participants. This approach guarantees that the classifier is strictly not influenced from the activity of the left-out subject, allowing generalization to unseen patients. The same precaution was applied to the behavioral measures. Specifically, each measure was z-scored (i.e., data were centered to have mean equals to zero and scaled to have standard deviation equals one) across all participants and sessions excluding the sessions of the left-out subject. The left-out subject's sessions were then normalized using the same mean and standard deviation used for z-scoring the other participants.

### 2.10 Robustness over sessions and patient populations

In order to test the stability of the behavioral and resting-state brain measures over day and sessions, we *i)* computed test-retest Pearson correlation between different days of recordings for each behavioral and resting-state measure separately; *ii)* built a two-class LDA classifier training it with the data of one day of recordings and testing it with the data of a different day for group #1, separately for brain and behavioral data. A classifier with high accuracy and AUC would indicate stable features over sessions.

In order to further test the generalizability of the classifier to unseen patients, we additionally built classifiers including only the patients of group #1 and we tested them in the patients of group #2. Two patients were included in both study groups (see Supplementary Table 1 for details). These patients were excluded from the training set (i.e., group #1) and used only for the testing set (i.e., group #2).

### 2.11 Correlation with spatial neglect

To investigate the potential clinical usability of the rsEEG topography-based patterns we assessed whether the latter could characterize different levels of spatial neglect as manifested in the SNT. For this aim, we first calculated a multivariate analysis of correlation, i.e., canonical correlation analysis (CCA), between the laterality indices (i.e., LI and F) and the coefficients of variation of each EEG-SVD topography and frequency band. For this analysis, we did not consider the healthy subjects. If we consider the CVs of the EEG-SVD components ($Y=(y_{1},\;\ldots ,\;y_{m})$ with m=20 – i.e., 4 frequency bands per 5 EEG-SVD components) and the laterality indices ($X=(x_{1},\;\ldots ,\;x_{n})$ with n=2 – i.e., LI and F) as vectors of random variables, and there are correlations among these variables, then the CCA would find linear combinations (i.e., canonical components) of the CVs and the laterality indices that have maximum correlation with each other. Specifically, the CCA seeks vectors $a\;\in \;R^{n}$ and $b\;\in \;R^{m}$ such that the random variables *a^T^X* and *b^T^Y* maximize the correlation $p=\text{corr}(a^{T}X,\;b^{T}Y)$. The significance of the found canonical components was tested by permutation test. Specifically, the CV of each EEG-SVD topography and frequency band were randomized over subjects and we then used CCA to find correlation between these surrogate data and the behavioral measures. We repeated this procedure 1000 times and took the 99th percentile of the resultant distribution of correlation as a significance threshold. Once identified the significant canonical components, the behavioral canonical scores (*U*) and the brain canonical scores (*V*) were obtained by projections over the canonical correlation vectors ($U=a^{T}X$ and $V=b^{T}Y$).

We then applied k-means cluster analysis (with k=3) to the behavioral canonical scores to subdivide patients in subgroups (i.e., no-, mild-, and severe-spatial neglect). The three clusters did not have the same number of patients and sessions (see Supplementary Table 10). We confirmed that the patients were correctly divided in subgroups characterized by different levels of spatial neglect by testing the differences between the subgroups in their pencil-and-paper test (PPT) scores, which were not used in the clustering process.

Finally, we deployed LDA to further test the ability of the CVs of the EEG-SVD topographies to discriminate patients with different levels of spatial neglect. For this, we excluded all the session of one patient at a time and we *i)* performed CCA between the laterality indices (i.e., LI and F) and the coefficients of variation of each EEG-SVD topography and frequency band as aforementioned; then *ii)* we applied k-means cluster analysis (with k=3) to the behavioral canonical scores to subdivide patients in subgroups (i.e., no-, mild-, and severe-spatial neglect). Finally, *iii)* we employed a three-class (i.e., no-, mild-, and severe-spatial neglect) LDA classifier (accounting for different covariance matrices for each class) to discriminate patients with different levels of spatial neglect. The class labels were assigned based on the subgroups of the k-means cluster. The features of the classifier (i.e., the CVs of the EEG-SVD components) were ranked using the absolute value of the brain canonical correlation coefficients of the first (i.e., the significant) canonical component. We sequentially added feature after feature and tested classification accuracy in the sessions of the left out patient. This three-step procedure was repeated for each patient (i.e., fold) and the decoding accuracy values were then averaged over cross-validation folds. Finally, we chose the combined classifier with the highest performance, and confusion matrices were computed considering all cross-validations folds.

## 3 Results

### 3.1 Patients are slower and less accurate than healthy subjects in visual search

**Fig. 1:**
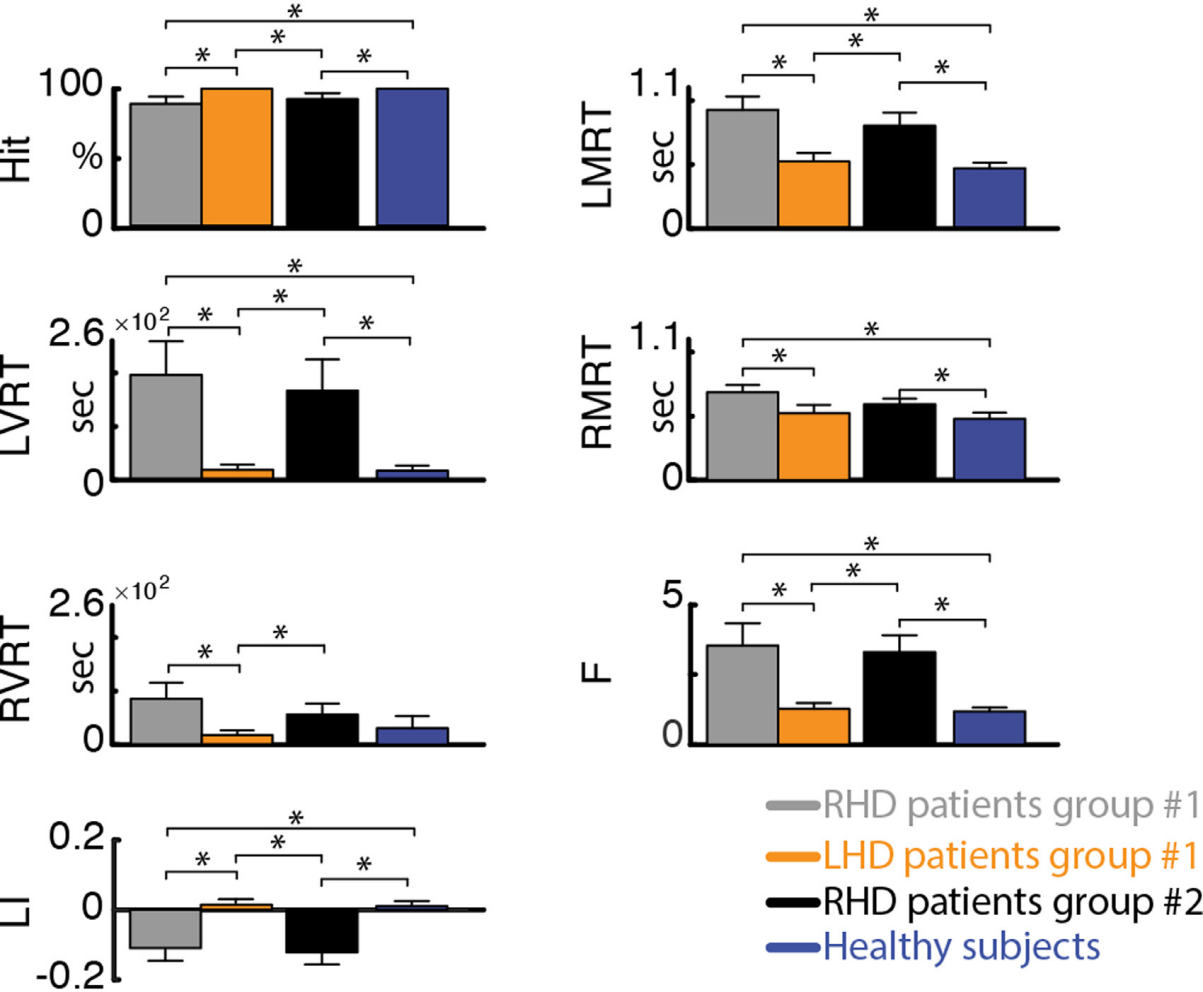
**Performance measures of the starry night test**. Hit – percent of targets detected out of all targets. LMRT, RMRT – mean reaction time to left- and right-sided targets, respectively. LVRT, RVRT – variance of reaction time for left- and right-sided targets, respectively. LI – laterality index, computed as $\text{LI}=\frac{\left(\text{RMRT}-\text{LMRT}\right)}{\left(\text{RMRT}+\text{LMRT}\right)}$. F – ratio of left and right coefficients of variability (i.e., $F=\frac{\text{LVRT}/|\text{LMRT}|}{\text{RVRT}/|\text{RMRT}|}$). Error bar represent average values +/− standard error of the mean (SEM) for group #1 for RHD patients (grey bars) and LHD patients (orange bars), for group #2 RHD patients (black bars), and for healthy subjects (blue bars). Significant differences between groups (one-tailed, non-paired *t*-test with heteroschedasticity (α=0.05)) are indicated with black asterisks.

Replicating our previous findings using the SNT (Deouell et al., 2005), RHD patients in both groups were significantly slower than healthy controls (see Fig. 1 and Supplementary Table 6). Moreover, RHD patients had longer reaction times in detecting left-sided compared to right-sided target stimuli (average ± standard error (SE) reaction times for group #1: LMRT = 919 ± 104 ms, RMRT = 695 ± 57 ms; for group #2: LMRT = 802 ± 99 ms, RMRT = 600 ± 47 ms). In contradistinction, healthy controls had similar reaction times for left- and right-sided target stimuli (average ± SE: LMRT = 470 ± 38 ms, RMRT = 484 ± 49 ms). This difference was reflected by a significantly more negative laterality index (LI) in RHD groups #1 and #2 (average ± SE: −0.11 ± 0.04 and −0.12 ± 0.03, respectively) compared to the control group (0.01 ± 0.01), and by a significantly higher F index for the RHD patients (group #1: 3.52 ± 0.83; group #2: 3.30 ± 0.62) versus controls (1.18 ± 0.14). The results were replicable across the patients groups and no differences were found between the RHD patients of the two groups (*p* > 0.37). In contradistinction, LHD patients were slightly but not significantly slower than healthy controls and did not display a significant effect of side (average ± SE: LMRT = 515 ± 64 ms, RMRT = 531 ± 64 ms; *p* > 0.16), congruent with the fact that spatial neglect is more severe and persistent following right than left brain damage (Corbetta and Shulman, 2011). Slightly slower RT for the LHD patients could be explained by the use of the left hand to perform the task despite their right hand dominance.

The test-retest correlation was high for both groups (average test-retest correlation across behaviors: 0.66 and 0.77 for group #1 and #2, respectively; Supplementary Table 7) indicating stability of the behavioral measures over days of recordings.

### 3.2 Spatially-distributed spectral sub-bands are reliable, and different in stroke patients and healthy subjects

To evaluate neural activity patterns post-stroke, we analyzed EEG activity at rest with eyes open using a 32-channels configuration. Like with the behavioral data, we evaluated the spectral sub-bands separately for RHD and LHD patients.

**Fig. 2:**
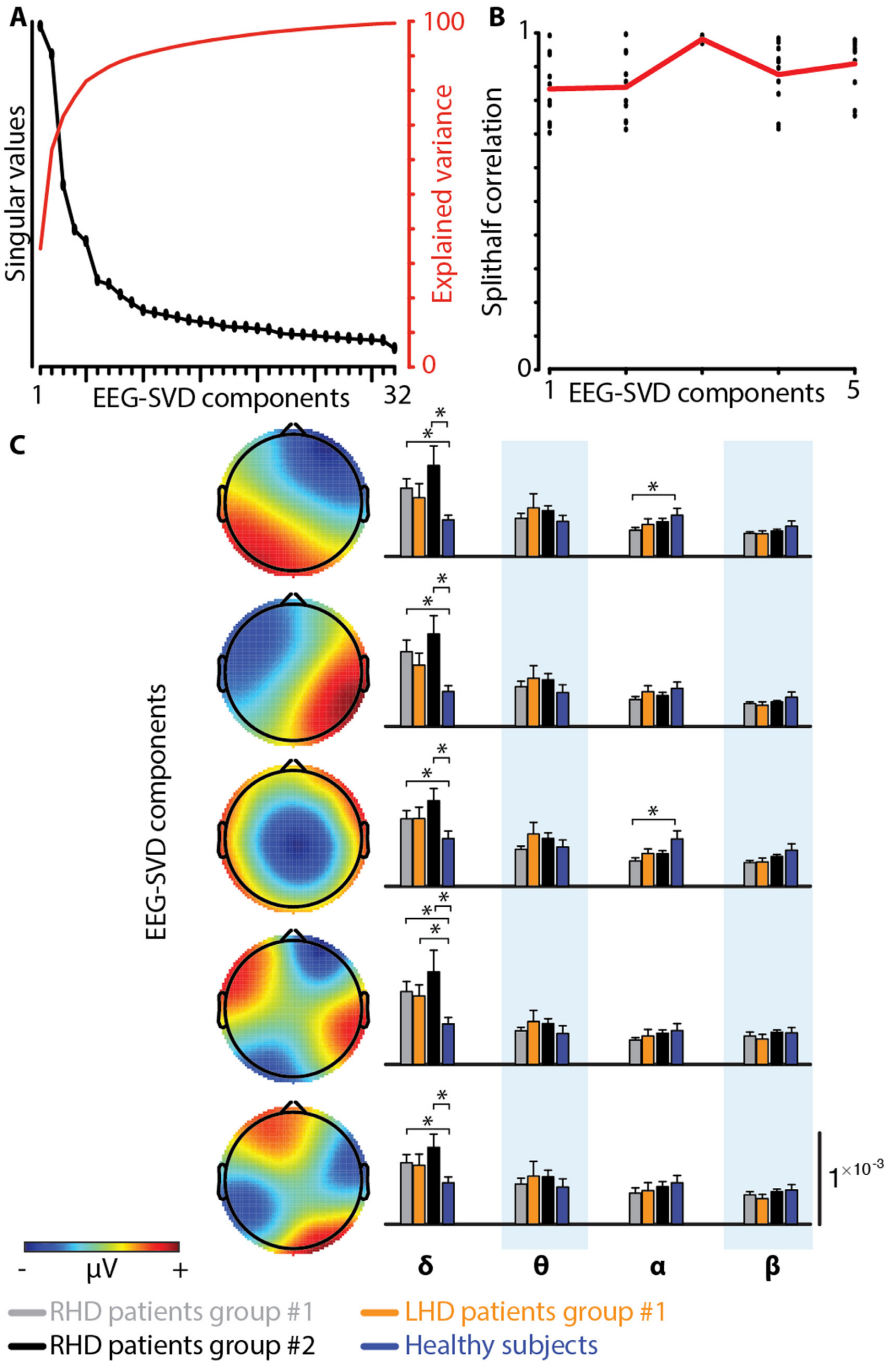
**rsEEG-SVD measures. (A)** Singular values and explained variance of the 32 EEG-SVD topographical components. The first 5 components were retained. **(B)** Split-half correlation of the first 5 components shows high reproducibility. **(C)** Each row represents one of the five EEG-SVD components, depicting the topography of the component on the left, and the coefficient of variations for the 4 frequency bands (δ: 1–3.8 Hz; θ: 4.1–7.6 Hz; α: 8.2–12.4 Hz; β: 15.3–30.6 Hz) on the right. Red and blue colors correspond to positive and negative voltages, respectively. Error bar represent average values +/− standard error of the mean (SEM) for group #1 for RHD patients (grey bars) and LHD patients (orange bars), for group #2 RHD patients (black bars), and for healthy subjects (blue bars). Significant differences between groups (one-tailed, non-paired *t*-test with heteroschedasticity (α=0.05)) are indicated with black asterisks.

We utilized SVD decomposition of the EEG signals to identify reliable and reproducible topographical maps (Sivakumar et al., 2016). We selected the first five EEG-SVD components, which accounted for 83% of the variance (Fig. 2A). We tested the consistency of the maps by split-half reproducibility analysis computed on the basis of 10 random splits (Fig. 2B). All the five EEG-SVD components were highly reproducible (average correlation ± standard deviation (STD) across splits and components: 0.89 ± 1.00).

Previous studies showed that compromised brain areas are characterized by an increase in power of the EEG lower spectral bands (delta and theta) and a decrease in the upper bands (alpha and beta) (Finnigan and van Putten, 2013). Spectral power coefficient of variations of the EEG-SVD components showed similar trends: higher coefficient of variations (CVs) in RHD patients than in healthy subjects for the delta band, and lower CVs for the alpha and beta bands (Fig. 2C). In particular, CVs for the delta band of all the five EEG-SVD components were significantly higher in RHD patients than in healthy subjects for patient group #1 (Supplementary Table 9). The CVs for the alpha band, instead, were significantly lower for the 1st and the 3rd component (*t* = 2.00; df = 6.75, *p* < 0.05 for 1st component and *t* = 2.52; df = 6.63, *p* < 0.02 for 3rd component). Significantly higher CVs for the delta band of all the five EEG-SVD components for RHD patients versus controls were replicable in patient group #2 (Supplementary Table 9).

The test-retest correlation values across days were higher than those of the computer-based behavioral measures (SNT results), with average test-retest correlation across frequency bands and EEG-SVD components equal to 0.87 and 0.79 for patient group #1 and #2, respectively, revealing a strong reliability of the resting-state EEG over days of recording (Supplementary Table 8).

### 3.3 Behavioral and rsEEG measures can be used to classify single patients vs. healthy controls

The previous results suggest that on average, the SNT measures as well as the EEG-SVD patterns distinguish RHD patients from healthy control subjects, and that these measures are stable at the group level, over repeated measurements. Next, we tested whether these measures could be robust enough to classify individual subjects over several days. For this, we *i)* designed a two-class LDA classifier collapsing across days of recordings behavioral measures and spontaneous brain activity separately and using leave-one-out approach; *ii)* tested the stability of the classifier when it is trained and tested over different days; and *iii)* tested the stability of the behavioral and electrophysiological measures over different patient populations building the classifier only with patients of group #1 (collapsing together day 1 and day 2) and testing it on patients of group #2.

**Fig. 3:**
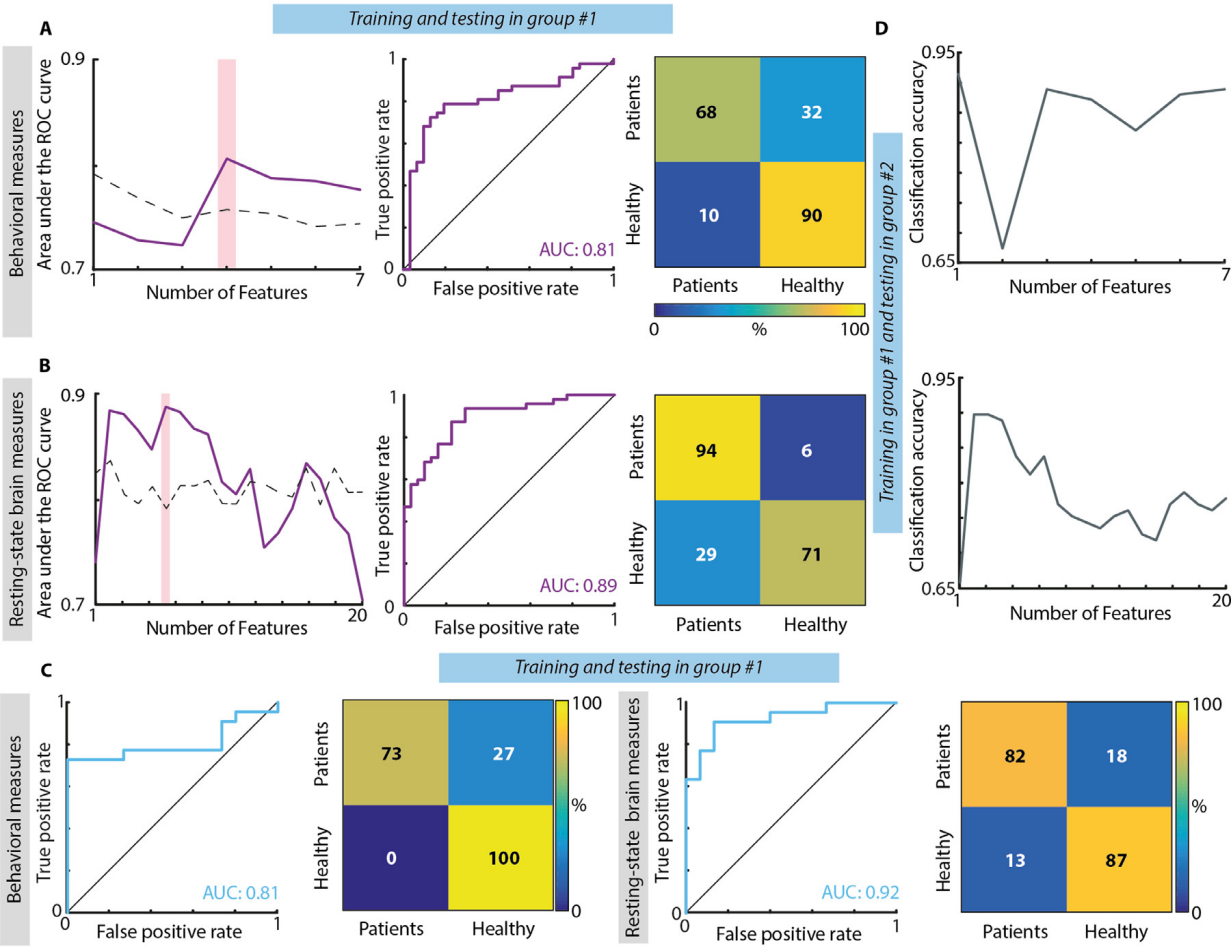
**Results of classification between RHD patients and controls. (A)** Classification based on behavioral measures of the SNT. Left: Area under the ROC curve with different number of features (purple line) and chance level based on permutation analysis (dashed line). The pink bar shows the optimal number of features selected (i.e., maximum AUC). Middle panel: ROC curve for the classifier obtained using the optimal number of features. Confusion matrix for patients and healthy controls for the classifier obtained using the optimal number of features. From top left clockwise: percent true positive, false negative, false positive, and true negative (hits, miss, false alarm and correct rejection, respectively). **(B)** Classification based on CVs of the five EEG-SVD components. The organization of the panel is the same used for the behavioral measures. (**C**) Classification results for the classifiers built training on the data of group #1 of day 1 and tested in the data of group #1 of day 2. Both for behavioral (1st and 2nd panels) and brain measures (3rd and 4th panels) we considered the features corresponding to the maximum AUC and we reported ROC curves (1st and 3rd panels) and confusion matrices (2nd and 4th panels). (**D**) Classification results for the classifiers built training on patients of group #1 and tested on patients of group #2. Top: classification accuracy with different number of features (grey line) for behavioral measures; bottom: classification accuracy with different number of features (grey line) for EEG-SVD measures.

When concatenating together both days of recordings for group #1, the highest classifier performance for the behavioral measures was obtained when considering together the laterality index, the mean and variance of reaction time for the left-sided targets, and mean reaction time for right-sided targets (Fig. 3A). These variables were able to distinguish between patients and healthy subjects with a high accuracy, stable over cross-validation folds, as also highlighted by a diagonal confusion matrix (maximum mean accuracy ± STD over cross-validation folds: 0.78 ± 0.32), and with a high sensitivity-specificity as emphasized by the ROC curve (AUC: 0.81 – null distribution threshold: 0.76). For resting-state brain activity, we found that the highest separation between patients and healthy subjects was achieved when combining the coefficients of variation of delta band for 1st, 2nd, and 5th EEG-SVD components, of alpha band for the 1st and 3rd component, and of beta band for the 3rd component (Fig. 3B). The discrimination accuracy and the sensitivity-specificity of the LDA classifier based on the EEG-SVD were high and stable over cross-validation folds (mean accuracy ± STD over cross-validation folds: 0.83± 0.35; AUC: 0.89 – null distribution threshold: 0.79).

To explore the stability of the behavioral and electrophysiological signatures over days of recordings, we built a two-class LDA classifier using the data of the first day of recordings of group #1 and testing it on those of the second day of recordings of the same patients. The AUC and the accuracy were high both for behavioral (mean accuracy ± STD over cross-validation folds: 0.81± 0.36; AUC: 0.81, Fig. 3C) and EEG-SVD measures (accuracy=0.81± 0.31 and AUC=0.92 Fig. 3C), highlighting the stability of these electrophysiological patterns and, thus, the potential of the spontaneous brain activity to serve as a tool for following long-term post-stroke outcome.

Both for behavioral and electrophysiological measures, the discrimination accuracy was high also when testing the classifier trained on patients of group #1 on patients of group #2 (accuracy: 0.87 and 0.81 for behavioral and brain measures, respectively – null distribution threshold: 0.71 both for behavioral measures and resting-state brain measures, Fig. 3D). This fact emphasizes the generalizability of these measures to new patients.

### 3.4 Resting-state EEG-SVD discriminates different levels of spatial neglect

**Fig. 4:**
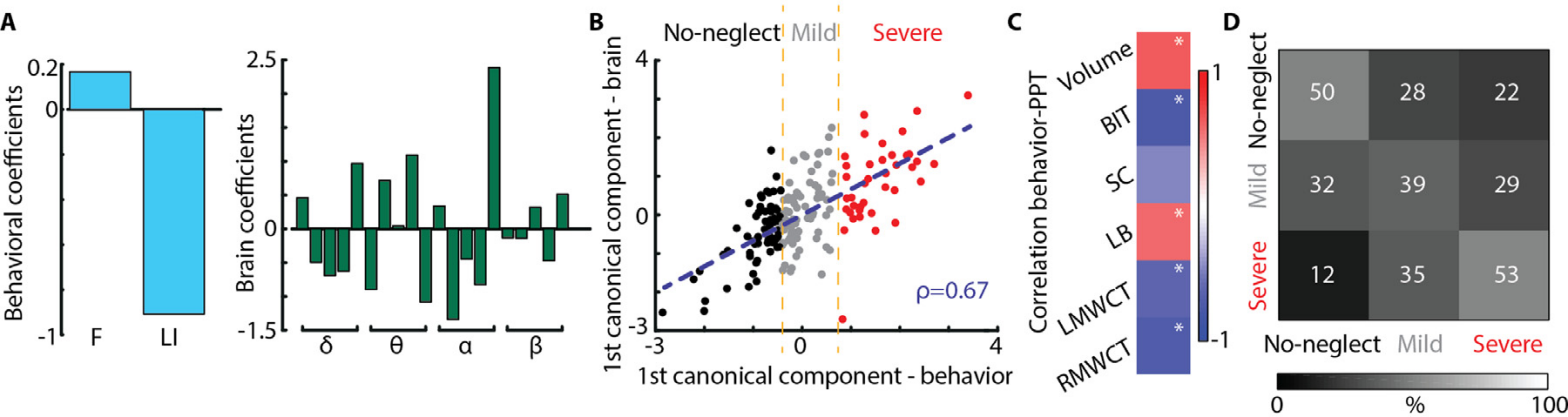
**Canonical correlation analysis between behavioral and CVs of EEG-SVD. (A)** Canonical correlation coefficients for laterality indices (i.e., LI and F) (left panel) and for CVs of the EEG-SVD components (right panel) for the first canonical component (i.e., significant component assessed by permutation test). The results reported in panels A-C relate to the CCA computed considering all the patients. **(B)** 2D correlation plot of canonical correlation scores for behavioral (x-axis) and brain measures (y-axis). Canonical correlation scores for behavioral measures clustered in 3 different clusters (no-neglect – black, mild-neglect – grey, and severe-neglect – red) using k-means algorithm. **(C)** Correlation between canonical correlation scores for behavioral measures and PPT scores for patients of group #2. **(D)** Confusion matrix for the 3-classes (no-, mild-, and severe-neglect) LDA classifier using leave-one-subject-out approach.

To further investigate the clinical potential of the identified rsEEG topography-based patterns, we assessed whether the coefficient of variations of the EEG-SVD components can discriminate different levels of spatial neglect. For this aim, we computed CCA between the SNT laterality indices and the EEG measures. We found one significant canonical correlation component (correlation: 0.67, *p* < 0.01 permutation test; Fig. 4B) with highest coefficients for LI and for CV of alpha band 2nd and 5th EEG-SVD components, theta band for 4th and 5th component, and delta band for 5th component (Fig. 4A). The behavioral canonical scores, obtained projecting the behavioral data over the canonical correlation vectors, correlated significantly with paper and pencil (PPT) tests of neglect and with the total lesion volume for group #2 in which we had complete data for these measures (Fig. 4C). Moreover, the behavioral canonical scores stably clustered into 3 patients subgroups characterized by different levels of spatial neglect (no-neglect, mild-neglect and severe-neglect) as demonstrated by the PPT scores (Fig. 4B and Supplementary Fig. 2A). Patients belonging to the severe spatial neglect cluster (i.e., cluster number 3) had a significantly lower performance in the behavioral inattention test (BIT; *t* = 12.75; df = 2.53, *p* < 0.0003 compared to cluster 1; *t* = 8.08; df = 7.83, *p* < 0.00003 compared to cluster 2) and in the Mesulam and Weintraub cancellation task as compared to the other two patients clusters (i.e., no- and mild spatial neglect) (*t* = 2.44; df = 2.92, *p* < 0.05 compared to cluster 1; *t* = 3.63; df = 7.10, *p* < 0.004 compared to cluster 2). The total BIT score as well as the star cancellation (SC) subtest were not significantly lower (*p* > 0.07) for the mild-neglect cluster (i.e., cluster number 2) as compared to no-neglect cluster (i.e., cluster number 1), yet the average scores of the mild-neglect cluster were below the cut-off for normality (129 points for the BIT (Wilson et al., 1987) and 52 for the SC subtest (Halligan et al., 1990)). Finally, LHD patients belonged to cluster number 1 (i.e., no-spatial neglect).

In order to further test the potential role of spatially-distributed spectral sub-bands in discriminating different levels of spatial neglect, we built a three-class LDA classifier. For that, we used the CVs of the sub-bands characterized by the strongest canonical correlation coefficients (Fig. 4A) as features and the labels of the k-means clusters for supervised learning using a leave-one-subject-out approach. The classifier was able to discriminate patients with different levels of spatial neglect as highlighted by a diagonal confusion matrix well above chance level (mean accuracy ± STD over classes: 0.47 ± 0.07, Fig. 4D – chance level: 0.33, Supplementary Figure 3A) and by a higher number of patients classified in the correct cluster as compared to the number of patients classified in the other two groups. Overall these results highlight a significant association between resting-state topography features and level of spatial neglect, offering the prospect of a new diagnostic tool for unilateral spatial neglect.

### 3.5 Comparison between a priori selected single EEG channels and EEG-SVD

In previous sections, we used topography-based analysis, which provides a global assessment without *a priori* hypothesis on a specific brain location, aiming to increase the sensitivity to abnormalities of brain regions that are not considered at the outset. However, because of the prevalence of parietal damage in patients affected by spatial neglect (Corbetta and Shulman, 2011), several previous studies considered as informative the theta and alpha power over the posterior parietal cortex, corresponding to the P3 and P4 electrodes in the 10–20 international EEG positioning system. Therefore, we assessed the discrimination ability of the P3 and P4 electrodes following the same approach employed for the topography-based analysis – i.e., *i)* CCA between the laterality indices and coefficients of variations of theta and alpha bands of P3 and P4 electrodes; *ii)* k-means clustering over the behavioral canonical scores; *iii)* LDA classifier to discriminate the three levels of spatial neglect using a leave-one-subject-out approach.

**Fig. 5:**
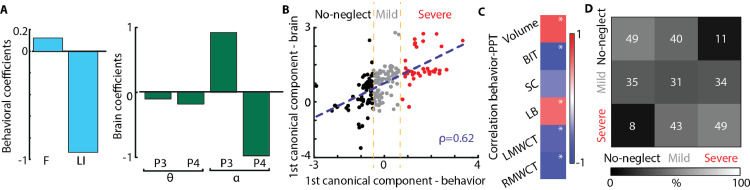
**Canonical correlation analysis between behavioral and CVs of P3 and P4 channels. (A)** Canonical correlation coefficients for laterality indices (i.e., LI and F) (left panel) and CVs of theta and alpha bands for the electrodes P3 and P4 (right panel) for the first canonical component (i.e., significant component assessed by permutation test). The results reported in panels A-C relate to the CCA computed considering all the patients. **(B)** 2D correlation plot of canonical correlation scores for behavioral measures (x-axis) and electrode spectrum (y-axis). Canonical correlation scores for behavioral measures clustered in 3 different clusters (no-neglect – black, mild-neglect – grey, and severe-neglect – red) using k-means algorithm. **(C)** Correlation between canonical correlation score for behavioral measures and PPT scores for patients of group #2. **(D)** Confusion matrix for the 3-classes (no-, mild-, and severe-neglect) LDA classifier using leave-one-subject-out approach.

As we found for the EEG-SVD topographies, here too we found one significant canonical correlation component (*p* < 0.01 permutation test; correlation: 0.62, Fig. 5C) with high coefficients for LI and for the coefficients of variation of alpha of the P4 and P3 electrodes (Fig. 5A). Similarly to the topography-based analysis, the behavioral canonical scores clustered in 3 patient subgroups characterized by different levels of spatial neglect (Supplementary Figure 2B). Indeed, the BIT and the SC subtest of the BIT were significantly lower for cluster number 3 (i.e., severe spatial neglect) as compared to cluster number 1 and 2 (BIT: *t* = 8.38; df = 1.34, *p* < 0.02 compared to cluster 1; *t* = 8.98; df = 9.00, *p* < 0.00004 compared to cluster 2; SC: *t* = 3.66; df = 2.99, *p* < 0.02 compared to cluster 1; *t* = 2.55; df = 5.05, *p* < 0.03 compared to cluster 2), and they were below the cut-off for normality for patients of cluster number 2 (mild spatial neglect). However, the three-class LDA classifier did not perform as well as the one constructed using the spatially-distributed spectral sub-bands (Fig. 5D). Patients with mild unilateral neglect were not distinguishable from the other 2 groups. Indeed, the percentage of mild-neglect patients correctly classified (0.31) was lower than the percentage of mild-neglect patients classified as no-neglect (0.35) or severe-neglect (0.34) patients (chance level: 0.33, Supplementary Fig. 3B).

## 4 Discussion

Current clinical decision-making in neurorehabilitation (e.g., decision on the specific therapeutic approach to be used for facilitating functional recovery in a given patient, or the decision to terminate an ongoing treatment based on its failure to demonstrate sufficient efficacy) lacks the benefit of credible biomarkers present in other clinical fields. Here, using repeated measurements in stroke patients and healthy controls, we found that resting-state EEG-SVD topographical patterns meet two critical criteria for useful biomarkers: *i)* robustness over repeated measurements and new patient samples (i.e., maintain an acceptable standard of reliability); and *ii)* correlation with the major clinical symptoms and signs of a given nosological entity, in a manner reflecting its severity and impact on patients’ life (i.e., maintaining an acceptable standard of specificity and sensitivity). EEG-SVD biomarkers allowed the detection of patients’ deviation from normality and allowed discriminating patients affected by different levels of unilateral spatial neglect in a reliable manner. We discuss these findings with an emphasis on the additional information garnered from EEG topography-based biomarkers, and their potential use in clinical practice of rehabilitation.

### 4.1 EEG recordings at rest are suitable for test-retest designs

Recently, a number of studies showed that features of resting-state brain activity can serve as reliable biomarkers, potentially aiding clinical decision making and treatment selection (Woo et al., 2017; Allali et al., 2018). Recordings of brain activity at rest may retain sensitivity for small but significant changes in high-functioning patients when ceiling effects prevent their demonstration by standard behavioral tests. In the current study we exploited this capacity and analyzed resting-state EEG activity with eyes open, to explore whether spontaneous brain activity patterns allow discrimination of brain-damaged patients from healthy controls, with preservation of sensitivity over repeated assessments and patient populations.

Selection of individual EEG channels as a data source for biomarkers aiming to discriminate patients with widespread neurological damage from healthy controls is tenuous, as the deficits are not necessarily identifiable by single-channel analysis, nor are the “critical” channels necessarily known in advance. Moreover, a general physiological feature of stroke-related impairment is the disruption of distributed network connectivity, both intra- and inter-hemispherical (Siegel et al., 2016). This requires coverage by a large set of electrodes to be captured. We therefore utilized topography-based analysis, which provides a more global interpretability without any a-priori hypothesis on the spatial location of abnormal brain activity.

Occurrence and duration of resting-state topographies have been previously shown to be sensitive to clinical changes induced by neurological and psychiatric disease (Allali et al., 2018; Serrano et al., 2018; Khanna et al., 2014; Michel and Koenig, 2017). Particularly, in stroke, preserved duration of resting-state topographies correlated with a better effective recovery (Zappasodi et al., 2017). In these studies, topographical maps (i.e., microstates) were obtained by temporal clustering and their time courses were computed by back-fitting of the EEG signals (Michel and Koenig, 2017). Here, instead, we employed SVD of the pre-processed signals of different subjects and sessions, which were concatenated across time. This concatenation allows for unique time courses for each subject, but assumes that all subjects have similar spatial map per component, which was indeed the case (see Supplementary Figure 1A-B). Temporal concatenation is particularly useful when neural fluctuations are expected to be different for different subjects, which it is often the case for resting-state activity (Calhoun et al., 2009; Beckmann et al., 2009; Tian et al., 2013). Moreover, the single temporal regression, as opposed to a dual spatiotemporal regression, allows to preserve the subject-specificity only on the time courses. This choice is justified by the EEG tradeoff between low/high spatial/temporal resolutions, which implies that subject-specific differences are captured mostly by temporal fluctuations. Other approaches have been proposed for concatenating subjects for group decomposition, as spatial concatenation, which allows for subject-specific maps but assumes common time courses, or higher order tensor decompositions, which estimate single spatial, temporal, and subject-specific ‘mode’ for each component (Chatzichristos et al., 2019). Yet these methods might work less well than temporal concatenation when the temporal variations across subjects are larger than the spatial variations, which is often the case in resting-state activity (Beckmann et al., 2009).

In our approach, time courses of individual maps were computed by projection of single-subject signals over the first five EEG-SVD components and were utilized to extract brain spectral signatures. Previous studies showed that the principal components of spontaneous neural activity are uniquely determined by the underlying circuit connections (Galán, 2008). EEG-SVD decomposition results in spatial topographies that are similar to the previously identified microstates (Michel and Koenig, 2017; Koenig et al., 2002) and spherical harmonics (Higashi et al., 2014; Sivakumar et al., 2016; Zare et al., 2018), which are a hallmark of spontaneous, large-scale synchronization of neural activity in the brain. SVD is usually deployed as a first dimensionality reduction step in independent component algorithms (ICA) (Calhoun et al., 2009), which further refine the components using (one of the proxies for) statistical independence. Eigenmodes from SVD can, therefore, be seen as a simpler and more straightforward decomposition method, as only second-order statistics are used. We used this measure here as we were not interested to investigate in-depth individual components, but rather the subspace spanned by the global ones.

We found that coefficients of variations of alpha bands of the EEG-SVD topographical maps were significantly lower in patients as compared to healthy subjects, whereas slower frequencies (delta) had higher spectral power in the patients. Excessive delta power after stroke has already been related to cognitive functioning (Aminov et al., 2017; Finnigan et al., 2016; Rijsdijk et al., 2008). Alpha and beta activity, instead, have often been shown to be lower in stroke participants than in age-matched adults, yet to date they have been considered less informative than other frequency bands for acute assessment and monitoring (Finnigan and van Putten, 2013).

Previous studies in healthy subjects showed that quantitative parameters of brain activity, such as spectral power of different bands, are intra-individually stable across repeated measurements (Laaksonen et al., 2013; Van Albada et al., 2007). We demonstrated here that these parameters did not significantly change across sessions also in brain-damaged individuals, as assessed by test-retest correlation across days, and linear discriminant analysis. Moreover, both spatial maps and their frequency contents were comparable across RHD patients of the two groups (see Supplementary Figure 1C). This finding shows that such parameters can be used in clinical practice, where a crucial requirement in longitudinal assessment aiming to quantify natural and treatment-induced recovery is to discriminate true improvement from non-significant test-retest variation, which does not reflect functional recovery.

### 4.2 EEG topography as a biomarker for unilateral spatial neglect

The LI and the F score of the SNT captured the spatial asymmetry in visual search, signaling USN. The coefficients of variations of theta and alpha bands of the EEG-SVD topographical maps were found to maintain a significant correlation with these behavioral measures, particularly with the LI. This finding is in line with recent results showing that impaired contralesional awareness of visual stimuli in USN is associated with reduced beta and theta activity in fronto-parietal attention networks (Yordanova et al., 2017; Fellrath et al., 2016). Reduced theta-band connectivity in the dorsal fronto-parietal network was found to correlate with impaired modulation of visual attention by goal-relevant distracters (Sacher et al., 2004).

Neglect is manifested in stroke patients most frequently following right temporo-parietal damage. In our cohort, alpha power at the P4 and P3 channels over the right parietal scalp was found to correlate with contralateral inattention as reflected in SNT performance. Yet, EEG-SVD topographies allowed a better discrimination of neglect patients. The higher sensitivity to the different levels of impairment results probably from the ability of the topographies to capture changes over several key cortical regions (attention network hubs), extending beyond the location of anatomical damage and contributing to the complex symptom of USN. EEG-SVD could, therefore, represent a valuable measure of the physiological dysfunction in the attention network that is the underlying mechanism behind USN phenomenology. As such, EEG-SVD could possibly serve to monitor the efficacy of treatment modalities aimed to improve the impaired physiology. Indeed, modulation of alpha and theta oscillations over the parietal cortex has been used in therapeutic interventions for USN based on EEG-neurofeedback (Ros et al., 2017; Saj et al., 2018; Hernandez et al., 2016; Sitaram et al., 2017) (for a recent review of EEG-neurofeedback see Sitaram et al., 2017). However, recent studies showed that feedback extracted from network-based measures, instead of single brain region features, could target more relevant large-scale interactions leading to potentially more powerful regulation protocols (Sitaram et al., 2017; Koush et al., 2017). This could be particularly relevant for USN, which represents a classical large-scale network dysfunction (Mesulam, 1999; Corbetta and Shulman, 2002), characterized by widespread changes of functional connectivity across different frequency bands extending far beyond the locus of structural brain damage (Corbetta and Shulman, 2011; Ros et al., 2019).

### 4.3 The SNT is stable across measurements and can be used to classify single patients

Computer-enhanced visual search tests like the SNT have been proven successful in capturing the essential contralesional disadvantage in information processing, manifested by USN patients. Moreover, these tests are more sensitive than traditional paper-and-pencil tests of neglect in capturing dynamics in USN severity (Bonato and Deouell, 2013). This higher sensitivity is conveyed by fine-grained quantitative measures that can be extracted from the continuously recorded information (i.e., reaction time and hit rate in their relation to target-stimulus position). Here, we evaluated whether this sensitivity is maintained over repeated tests and patient populations, providing the basis for longitudinal patients’ assessments. For this aim, we considered patients belonging to two different groups. In both groups, RHD patients responded more slowly and less accurately than LHD patients and healthy control subjects, in particular for left-sided target stimuli, thus manifesting the essential features of USN in an easily quantifiable manner. (Corbetta and Shulman, 2011; Bonato, 2012). Adding to previous reports of the SNT, linear discriminant analysis based on SNT features allowed distinguishing between RHD patients and healthy subjects with high sensitivity and specificity. The discrimination accuracy generalized to an unseen cohort of RHD patients (i.e., from group #1 to group #2). Moreover, the sensitivity to the spatial deficits was preserved during the second day of recordings as also highlighted by high classification accuracy when training and testing the classifier in different days. The retained sensitivity was probably afforded by the unpredictable nature of target location in the test, which deploy attentional monitoring resources and reduce the implementation of compensatory strategies (Bonato and Deouell, 2013).

## 5 Conclusion

We showed that resting-state EEG-SVD measures allow a good sensitivity-specificity in discriminating patients’ brain activity from normal activity, in a highly heterogeneous (in terms of impairment severity and lesion location) group of stroke patients. Discrimination accuracy was preserved over repeated measures, supporting the usability of resting-state EEG-SVD as biomarkers for longitudinal assessments. Moreover, spatially-distributed spectral sub-bands of the EEG were found to correlate with side differences in reaction time to target stimuli in a visual-search task, thus allowing to discriminate patients with USN of different severity levels. We suggest that analysis of resting-state EEG topography may provide insight into the physiological status of a patient and its modulation by treatment, providing important additional data on which clinical decision-making can rely.

## CRediT authorship contribution statement

**Elvira Pirondini:** Conceptualization, Methodology, Software, Formal analysis, Visualization, Writing - original draft. **Nurit Goldshuv-Ezra:** Investigation, Data curation. **Nofya Zinger:** Investigation, Data curation. **Juliane Britz:** Funding acquisition. **Nachum Soroker:** Funding acquisition, Supervision, Writing - review & editing, Resources, Conceptualization, Project administration. **Leon Y. Deouell:** Funding acquisition, Project administration, Supervision, Writing - review & editing, Validation, Conceptualization. **Dimitri Van De Ville:** Funding acquisition, Supervision, Writing - review & editing, Conceptualization, Methodology.

## Appendix Supplementary materials

Image, application 1

## Footnotes

2 Biomarker - A characteristic that is objectively measured and evaluated as an indicator of normal biologic processes, pathogenic processes, or pharmacologic responses to a therapeutic intervention 12 Group, B. D. W. et al. Biomarkers and surrogate endpoints: preferred definitions and conceptual framework. *Clinical pharmacology & therapeutics***69**, 89-95 (2001).
