# Supplementary material for: Resting-state EEG topographies: Reliable and sensitive signatures of unilateral spatial neglect
Source: Neuroimage Clin. 2020 Mar 5;26:102237. doi: 10.1016/j.nicl.2020.102237 (PMC7083886; doi:10.1016/j.nicl.2020.102237)
Supplement: Supplementary file 1 [file mmc1.docx]

**Supplementary materials for manuscript: Resting-State EEG Topographies: Reliable and Sensitive Signatures of Unilateral Spatial Neglect**

**Supplementary Material**

In order to demonstrate that all subjects had similar spatial map per component, we first performed SVD decomposition of the EEG signal of each participant and every session. The spatial maps obtained for each participant and session were matched to the spatial maps obtained concatenating all subjects and sessions (i.e., group-level EEG-SVD maps) using Hungarian algorithm[^1^](#_ENREF_1). We computed correlation values between the individual spatial maps and the group-level EEG-SVD maps. The correlation was high for all top-five components (mean and standard deviation across components: 0.77± 0.10 – see Supplementary Figure 1A) highlighting that all the subjects had similar spatial maps. Second, to further support the similarity across the topographies of the individual subjects, we computed a spatial regression to acquire subject-specific spatial maps. Specifically, we ran the time-concatenated SVD (i.e., concatenating all subjects and sessions) and then we regressed per subject the spatial maps of the component; i.e., we used each subject/session part of the components’ time-courses as regressors against this subject/session data to retrieve subject-level spatial maps. We then compute the correlation between these subject-level spatial maps with the group-level EEG-SVD maps obtained concatenating all subjects and sessions. The correlation was high for all top-five components (mean and standard deviation across components: 0.84 ± 0.12 – see Supplementary Figure 1B) highlighting that all the subjects had similar spatial maps and also similar to the group-level EEG-SVD topographies.

**Supplementary figures**

**Supplementary figure 1: (A)** Correlation between individual maps and group-level EEG-SVD maps for the first five components. Error bars represent average values +/− standard error of the mean (SEM) across participants. **(B)** Correlation between subject-level maps (i.e., obtained with spatial regression) and group-level EEG-SVD maps for the first five components. Error bars represent average values +/− standard error of the mean (SEM) across participants. **(C)** We computed EEG-SVD decomposition concatenating separately RHD patients of group #1 and RHD patients of group #2. Bars represent correlation between EEG-SVD group-level topographical maps for RHD patients of group #1 and EEG-SVD maps for RHD patients of group #2.

**Supplementary figure 2:** **Paper and pencil scores** divided for no-neglect, mild, and severe spatial neglect patients identified using k-means clustering algorithm. Error bars represent average values +/− standard error of the mean (SEM) for no-neglect (black bars), mild-neglect (grey bars), and severe neglect (red bars) patients. When available we report the cut-off for spatial neglect. **(A)** The patients were clustered using the behavioral canonical correlation scores obtained from CCA between behavior and CVs of the EEG-SVD components. **(B)** The patients were clustered using the behavioral canonical correlation scores obtained from CCA between behavior and CVs for theta and alpha bands for the P3 and P4 electrodes.

**Supplementary figure 3: Chance level confusion matrix** for the three-class classifier built using the CVs of the EEG-SVD components **(A)** and for the three-class classifier built using the CVs for theta and alpha bands for the P3 and P4 electrodes **(B)**.

**Supplementary tables**

**Table 1**: summary of stroke patients enrolled in the study

|  | **Patients** | **Gender** | **Age yrs** | **Days elapsed**  **from the accident** | **Dominance** | **Location** |
| --- | --- | --- | --- | --- | --- | --- |
| **Group #1** | p101 | M | 66 | 78 | R | Right MCA - TP |
|  | p102 | M | 44 | 35 | R | Left IC, BG, ACT |
|  | p103 | F | 59 | 21 | R | Left CP, IHWM |
|  | p104 | M | 70 | 22 | R | Right MCA- FP |
|  | p105 | M | 79 | 57 | R | Left MCA - TP |
|  | p106 | F | 51 | 32 | R | Right MCA - EC, INS |
|  | p107 | M | 68 | 25 | R | Right MCA - Th, IC |
|  | p108 | M | 67 | 26 | R | Right MCA - TP |
|  | p109 | M | 77 | 91 | R | Right MCA - F |
|  | p110 | M | 47 | 21 | L | Right MCA - FTP |
|  | p111 | M | 63 | 34 | R | Right MCA – IC, BG |
|  | p112 | F | 62 | 32 | R | Left MCA – IC, BG |
|  | p113 | M | 54 | 71 | R | Right MCA – TH, IC |
|  | p114 | M | 56 | 60 | R | Right MCA – TH, EC |
|  | p115 | M | 53 | 48 | R | Right PCA, MCA – TO |
|  | p116 | F | 70 | 45 | R | Right MCA – TP, IC, BG |
|  | p117 | F | 65 | 39 | R | Left MCA |
|  | p118 | M | 77 | 43 | R | Right MCA – FTP |
|  | p119 | M | 54 | 52 | R | Right MCA – Caudate |
|  | p120 | M | 60 | 26 | R | Right MCA – F |
|  | p121 | M | 46 | 84 | R | Right MCA |
| **Group #2** | p201 | F | 67 | 54 | R | Right MCA – FP |
|  | p202 | M | 66 | 45 | R | Right MCA – FTP |
|  | p203 | M | 71 | 60 | R | Right MCA – FTP |
|  | p204 | M | 63 | 34 | R | Right MCA – FTP |
|  | p205 | F | 54 | 13 | R | Right MCA – CP |
|  | p206 | M | 66 | 61 | R | Right MCA – P |
|  | p207 | M | 72 | 29 | R | Right MCA – FP |
|  | p208 | M | 67 | 26 | R | Right MCA – TP |
|  | p209 | M | 45 | 61 | R | Right MCA – FTP |
|  | p210 | F | 44 | 20 | R | Right MCA – FTP |
|  | p211 | M | 68 | 70 | R | Right MCA – P |
|  | p212 | M | 53 | 60 | R | Right MCA – FTP |
|  | p213 | F | 58 | 77 | R | Right MCA – FTP |

Labels in the 2^nd^ column refer to (F) Female and (M) Male. Labels in the 5^th^ column refer to (L) Left and (R) Right. Labels in the 6^th^ column refer to (RHD and LHD) right and left hemisphere damage, respectively; (MCA) middle cerebral artery; F/P/T/O - Frontal/ Parietal/ Temporal/ Occipital, respectively; (STG) superior temporal gyrus; (IPL) inferior parietal lobule; (BG) basal ganglia; (IC) internal capsule; (EC) External capsule; (INS) insula; (IHWM) intra-hemishperic white matter; (TH) Thalamus. Subjects highlighted in grey were excluded from the analysis because unable to complete any of the sessions of recordings (i.e., resting-state and SNT task). The subject highlighted in pink was excluded from the analysis because left-handed. Subjects highlighted in orange were recorded in both studies.

**Table 2**: Paper and pencil test results and volume lesion for patients of group #2

|  | **Patients** | **Lesion volume loss (cm^3^)** | **BIT** | **SC** | **LB** | **MWCT L(/30)** | **MWCT R(/30)** |
| --- | --- | --- | --- | --- | --- | --- | --- |
| **Group #2** | p201 | 30.92 | 135 | 52 | 14.5 | 25 | 30 |
|  | p202 | 90.57 | 133 | 51 | 13.8 | 26 | 30 |
|  | p203 | 169.93 | 63 | 35 | 38.0 | 0 | 14 |
|  | p204 | 96.64 | 95 | 30 | 23.2 | 0 | 13 |
|  | p205 | 20.77 | 125 | 46 | 10.5 | 3 | 16 |
|  | p206 | 92.41 | 135 | 52 | 10.1 | 22 | 24 |
|  | p207 | 19.33 | 135 | 52 | 17.0 | 29 | 30 |
|  | p208 | 129.95 | 106 | 32 | 8.7 | 15 | 19 |
|  | p209 | 59.21 | 71 | 19 | 11.8 | 7 | 14 |
|  | p210 | 19.12 | 139 | 53 | 12.1 | 16 | 23 |
|  | p211 | 29.39 | 128 | 52 | 3.8 | 26 | 28 |
|  | p212 | 199.79 | 72 | 30 | 31.4 | 0 | 8 |
|  | p213 | 54.8 | 121 | 29 | 11.8 | 8 | 20 |

**Table 3**: summary of the recordings sessions for healthy subjects and patients of group #1

|  | **Subjects** | **Day 1** | | | **Day 2** | | | **Days between D1 and D2** |
| --- | --- | --- | --- | --- | --- | --- | --- | --- |
|  |  | **Morning** | **Afternoon** | **Evening** | **Morning** | **Afternoon** | **Evening** |  |
| **Healthy subjects** | c301 |  |  |  |  |  |  | 7 |
|  | c302 |  |  |  |  |  |  | 5 |
|  | c303 |  |  |  |  |  |  | -- |
|  | c304 |  |  |  |  |  |  | 7 |
|  | c305 |  |  |  |  |  |  | 7 |
|  | c306 |  |  |  |  |  |  | 7 |
| **Patients group #1** | p101 |  |  |  |  |  |  | 7 |
|  | p102 |  |  |  |  |  |  | 1 |
|  | p104 |  |  |  |  |  |  | 1 |
|  | p105 |  |  |  |  |  |  | 43 |
|  | p106 |  |  |  |  |  |  | 11 |
|  | p107 |  |  |  |  |  |  | 7 |
|  | p108 |  |  |  |  |  |  | 7 |
|  | p109 |  |  |  |  |  |  | -- |
|  | p111 |  |  |  |  |  |  | 7 |
|  | p112 |  |  |  |  |  |  | 5 |
|  | p114 |  |  |  |  |  |  | 2 |
|  | p115 |  |  |  |  |  |  | -- |
|  | p116 |  |  |  |  |  |  | 2 |
|  | p117 |  |  |  |  |  |  | 7 |
|  | p118 |  |  |  |  |  |  | 9 |
|  | p119 |  |  |  |  |  |  | 7 |
|  | p120 |  |  |  |  |  |  | 1 |
|  | p121 |  |  |  |  |  |  | -- |

Green squares indicate that an entire session (i.e., resting-state and SNT task) was completed and included in the analysis.

**Table 4**: summary of the recordings sessions for patients of group #2. Days between sessions are reported as mean ± std over following sessions.

|  | **Subjects** | **Days of recordings** | | | | | | | | | | **Days between sessions** |
| --- | --- | --- | --- | --- | --- | --- | --- | --- | --- | --- | --- | --- |
|  |  | **D1** | **D2** | **D3** | **D4** | **D5** | **D6** | **D7** | **D8** | **D9** | **D10** |  |
| **Patients group #2** | p201 |  |  |  |  |  |  |  |  |  |  | 4 ± 2 |
|  | p202 |  |  |  |  |  |  |  |  |  |  | 3 ± 1 |
|  | p203 |  |  |  |  |  |  |  |  |  |  | 2 ± 1 |
|  | p204 |  |  |  |  |  |  |  |  |  |  | 2 ± 1 |
|  | p205 |  |  |  |  |  |  |  |  |  |  | 3 ± 3 |
|  | p206 |  |  |  |  |  |  |  |  |  |  | 2 ± 1 |
|  | p207 |  |  |  |  |  |  |  |  |  |  | 1 ± 1 |
|  | p208 |  |  |  |  |  |  |  |  |  |  | 2 ± 1 |
|  | p209 |  |  |  |  |  |  |  |  |  |  | 2 ± 1 |
|  | p210 |  |  |  |  |  |  |  |  |  |  | 2 ± 1 |
|  | p211 |  |  |  |  |  |  |  |  |  |  | 2 ± 2 |
|  | p212 |  |  |  |  |  |  |  |  |  |  | 2 ± 1 |
|  | p213 |  |  |  |  |  |  |  |  |  |  | 1 ± 1 |

Green squares indicate that an entire session (i.e., resting-state and SNT task) was completed and included in the analysis.

**Table 5**: Percentage of outliers for healthy control (first column), LHD patients (second column), RHD patients of group #1 (third column), and RHD patients of group #2 (fourth column). Values indicate mean ± SEM over subjects. We compared the number of outliers between healthy subjects and *i)* LHD, *ii)* RHD patients of group #1, and *iii)* RHD patients of group #2 (one-tailed, non-paired t-test with heteroschedasticity (α=0.05)). No significant differences were found.

|  | **Healthy**  **subjects** | **LHD** | **RHD**  **group #1** | **RHD**  **group #2** |
| --- | --- | --- | --- | --- |
| **%Outliers** | 2.08  ±0.20 | 0.82  ±0.31 | 1.72  ±0.26 | 2.08  ±0.16 |

**Table 6**: p-values, degree of freedom of the test, and t-value of the statistical test comparing RHD patients and healthy controls for the behavioral measures for the two groups.

|  |  | Hit | LMRT | LVRT | RMRT | RVRT | F | LI |
| --- | --- | --- | --- | --- | --- | --- | --- | --- |
| **Group #1** | p-value  df  t | 0.02  13.00  2.23 | 0.0003  15.74  4.21 | 0.006  13.50  2.94 | 0.004  16.62  3.00 | 0.07  17.67  1.53 | 0.006  13.69  2.90 | 0.003  15.35  -3.15 |
| **Group #2** | p-value  df  t | 0.04  12.00  1.97 | 0.003  14.83  3.29 | 0.013  12.49  2.55 | 0.04  14.00  1.83 | 0.19  13.95  0.91 | 0.002  13.12  3.49 | 0.0002  15.54  -4.50 |

**Table 7**: test-retest correlation values for behavioral measures for patients of group #1 and group #2. (*) indicate significant correlation. For patients of group #2 the test-retest correlation was calculated between days that had all the 13 patients included in the analysis (i.e., day 1, day 3, and day 7). Values indicate mean±std over pair of days.

|  | **Hit** | **LMRT** | **LVRT** | **RMRT** | **RVRT** | **F** | **LI** |
| --- | --- | --- | --- | --- | --- | --- | --- |
| **Group #1** | 0.55 | 0.82 (*) | 0.61 (*) | 0.91 (*) | 0.27 | 0.62 (*) | 0.85 (*) |
| **Group #2** | 0.94±  0.04(*) | 0.95±  0.03(*) | 0.90±  0.04(*) | 0.80±  0.09(*) | 0.67±  0.19(*) | 0.30±  0.12 | 0.86±  0.09(*) |

**Table 8**: test-retest correlation values for resting-state brain measures for patients of group #1 and group #2. (*) indicate significant correlation. For patients of group #2 the test-retest correlation was calculated between days that had all the 13 patients included in the analysis (i.e., day 1, day 3, and day 7). Values indicate mean±std over pair of days.

|  | Frequency band | 1^st^ | 2^nd^ | 3^rd^ | 4^th^ | 5^th^ |
| --- | --- | --- | --- | --- | --- | --- |
| **Group #1** | δ | 0.96 (*) | 0.92 (*) | 0.92 (*) | 0.92 (*) | 0.97 (*) |
|  | θ | 0.82 (*) | 0.91 (*) | 0.79 (*) | 0.93 (*) | 0.87 (*) |
|  | α | 0.70 (*) | 0.92 (*) | 0.79 (*) | 0.68 (*) | 0.82 (*) |
|  | β | 0.95 (*) | 0.93 (*) | 0.77 (*) | 0.96 (*) | 0.91 (*) |
| **Group #2** | δ | 0.95 ± 0.03 (*) | 0.95 ± 0.03 (*) | 0.89 ± 0.07 (*) | 0.90 ± 0.06 (*) | 0.91 ± 0.06 (*) |
|  | θ | 0.84 ± 0.10(*) | 0.88 ± 0.05 (*) | 0.85 ± 0.07 (*) | 0.79 ± 0.09 (*) | 0.85 ± 0.04 (*) |
|  | α | 0.55 ± 0.23 (*) | 0.83 ± 0.05 (*) | 0.78 ± 0.10 (*) | 0.57 ± 0.18 (*) | 0.80 ± 0.09 (*) |
|  | β | 0.79 ± 0.01 (*) | 0.72 ± 0.13 (*) | 0.78 ± 0.06 (*) | 0.54 ± 0.11 (*) | 0.67 ± 0.19 (*) |

**Table 9**: p-values, degree of freedom of the test, and t-value of the statistical test comparing RHD patients and healthy controls for the CVs of delta band for the five EEG-SVD components and the two groups.

|  | Freq. band |  | 1^st^ | 2^nd^ | 3^rd^ | 4^th^ | 5^th^ |
| --- | --- | --- | --- | --- | --- | --- | --- |
| **Group #1** | δ | p-value  df  t | 0.004  17.04  -3.06 | 0.003  17.29  -3.08 | 0.048  15.29  -1.78 | 0.008  17.99  -2.69 | 0.028  17.88  -2.04 |
| **Group #2** | δ | p-value  df  t | 0.009  13.10  -2.70 | 0.005  14.04  -3.00 | 0.009  16.98  -2.65 | 0.018  13.98  -2.31 | 0.013  15.39  -2.47 |

**Table 10**: Number of patients (and sessions) per cluster. For the sessions we reported the mean and standard deviation over the patients of the cluster.

|  | **No-neglect** | **Mild-neglect** | **Severe-neglect** |
| --- | --- | --- | --- |
| **Group #1** | 11 (4±2) | 9 (2±1) | 8 (1.2±0.5) |
| **Group #2** | 10 (3±2) | 12 (5±2) | 5 (6±3) |

**Reference**

1 Munkres, J. Algorithms for the assignment and transportation problems. *Journal of the society for industrial and applied mathematics* **5**, 32-38 (1957).
